# Loss of diversity in wood-inhabiting fungal communities affects decomposition activity in Norway spruce wood

**DOI:** 10.3389/fmicb.2014.00230

**Published:** 2014-05-20

**Authors:** Lara Valentín, Tiina Rajala, Mikko Peltoniemi, Jussi Heinonsalo, Taina Pennanen, Raisa Mäkipää

**Affiliations:** ^1^Vantaa Research Unit, Finnish Forest Research InstituteVantaa, Finland; ^2^Department of Chemical Engineering, Technical School of Engineering, Universitat Autònoma de BarcelonaBarcelona, Spain; ^3^Department of Food and Environmental Sciences, University of HelsinkiHelsinki, Finland

**Keywords:** biodiversity, woody debris, respiration activity, functional redundancy, enzymes

## Abstract

Hundreds of wood-inhabiting fungal species are now threatened, principally due to a lack of dead wood in intensively managed forests, but the consequences of reduced fungal diversity on ecosystem functioning are not known. Several experiments have shown that primary productivity is negatively affected by a loss of species, but the effects of microbial diversity on decomposition are less studied. We studied the relationship between fungal diversity and the *in vitro* decomposition rate of slightly, moderately and heavily decayed *Picea abies* wood with indigenous fungal communities that were diluted to examine the influence of diversity. Respiration rate, wood-degrading hydrolytic enzymes and fungal community structure were assessed during a 16-week incubation. The number of observed OTUs in DGGE was used as a measure of fungal diversity. Respiration rate increased between early- and late-decay stages. Reduced fungal diversity was associated with lower respiration rates during intermediate stages of decay, but no effects were detected at later stages. The activity of hydrolytic enzymes varied among decay stages and fungal dilutions. Our results suggest that functioning of highly diverse communities of the late-decay stage were more resistant to the loss of diversity than less diverse communities of early decomposers. This indicates the accumulation of functional redundancy during the succession of the fungal community in decomposing substrates.

## Introduction

The loss of species diversity alters ecosystem function, stability and the ability to provide goods and services to society (Loreau et al., 2001; Hooper et al., 2005; Wertz et al., 2006; Cardinale et al., 2012). Several experiments have shown that primary productivity is negatively affected by a loss of plant species (Loreau et al., 2001; Hooper et al., 2012) and ecosystem stability is reduced by decreasing functional diversity (Hooper et al., 2005). Biodiversity affects the rate of key ecosystem processes such as decomposition and nutrient cycling (Loreau et al., 2001; Hättenschwiler et al., 2005; Gessner et al., 2010). In boreal forests, where fungal communities are major decomposers (Rayner and Boddy, 1988; Lindahl and Boberg, 2008; Stenlid et al., 2008), a high diversity of soil fungi can enhance the decomposition rate (Tiunov and Scheu, 2005), especially under environmental fluctuations such as variations in temperature regimes (Toljander et al., 2011). Hundreds of fungal species in Fennoscandian forests are now threatened, principally due to a lack of dead wood in a forest landscape that is intensively managed (Siitonen, 2001). The consequences of reduced fungal diversity on decomposition are unknown. Furthermore, the stability and function of forest ecosystems might be dramatically affected if the fungal capacity to resist habitat perturbation is continuously exceeded, as is the case for many species in managed forests (Stenlid et al., 2008).

Since fungal species produce complementary enzymes, the presence of many species can improve the communities' efficiency to degrade a wide range of litter constituents and thus enhance decomposition rate (Gessner et al., 2010). Experiments with selected fungal species and their combinations revealed a strong positive relationship between fungal diversity and decomposition rate, which became asymptotic at a relatively low level of diversity (Setälä and McLean, 2004; Tiunov and Scheu, 2005). In experiments involving a few species, facilitation and resource partitioning have been observed (Tiunov and Scheu, 2005). In more diverse terrestrial fungal communities, antagonistic mechanisms might prevail (Boddy, 2000; Gessner et al., 2010) and the colonization sequence of wood-degrading fungi can further affect decomposition (Fukami et al., 2010). Studies of the decomposition rate in manipulated fungal communities are mostly performed with a few cultured species, and the results might be a poor reflection of natural communities, especially if certain community members have a greater proportional influence on the decomposition rate than the totality of the fungal community (Robinson et al., 2005).

Decaying Norway spruce logs harbor diverse fungal communities (Ovaskainen et al., 2010; Rajala et al., 2011; Kubartová et al., 2012), where the number of active species is far higher than that considered in experimental studies. Species number tends to increase with mass loss (and related changes in the substrate quality) and peaks in the most decayed logs (Rajala et al., 2012). Decomposition of cell wall polymers in wood is a complex process that is driven by a succession of species with different litter-degrading enzymes (Baldrian, 2008; Stenlid et al., 2008). During the decay process of dead wood over several decades, the fungal decomposer community changes from one dominated by ascomycetes to one of white- and brown-rot basidiomycetes, before slowly becoming one composed mainly by the mycorrhizal species found in the underlying soil (Rajala et al., 2010, 2012). Wood-degrading fungi (white-rot and brown-rot fungi) decompose polysaccharides by producing hydrolases, but only white-rot fungi efficiently degrade lignin via oxidative metalloenzymes (e.g., laccase and manganese peroxidase) (Lundell et al., 2010). Experiments with decomposing leaves and needles have linked enzyme activities (EA) with fungal communities (Šnajdr et al., 2011; Žifèáková et al., 2011), but to our knowledge, no study has yet analyzed such a relationship with the fungal community in decomposing wood. Therefore, we studied wood substrates in different stages of decay to examine the effects of manipulated fungal diversity on enzyme production and decomposition rate during the entire process.

The overall objective of this study was to examine the relationship between fungal diversity and decomposition rate by manipulating fungal communities obtained from decaying Norway spruce wood. We designed a microcosm experiment to investigate: (1) whether a reduced diversity of wood-inhabiting fungi affects the decomposition of Norway spruce wood and, if so, (2) whether functional redundancy and stability of the decomposition process varies among decay stages, and (3) whether changes in fungal diversity and rate of decomposition are related to changes in hydrolytic enzyme production. We tested the hypothesis that CO_2_ production and enzyme activity in the dead wood are affected by the decay stage of the substrate and diversity of the decomposing species. Furthermore, we hypothetisized that the decomposition activity (measured as CO_2_ production) is correlated to overall variation of the fungal community and the decomposition increases to direction that is parallel to increasing species diversity (measured as number of detected OTUs).

## Materials and methods

### Wood samples

The study material was collected from an unmanaged forest in Lapinjärvi (Southern Finland, 60°39.413′N, 26°7.352′E, altitude 50 m, temperature sum 1300°C d; further details are provided in Rajala et al., 2012). Wood samples were obtained from stem discs sawn on site from 46 fallen Norway spruce (*Picea abies*) logs (diameter >5 cm at breast height). Logs were classified as early, intermediate or late stages of decay (I, III, and V according to Mäkinen et al., 2006). Sample discs were sawn in May 2011 and stored in plastic bags at −18°C until processing in the laboratory. The properties of the wood (C/N ratio, lignin content and density) for individual discs were analyzed as described in Rajala et al. (2010) and the mean values of 14 discs from early-decay stages, 12 discs from intermediate-decay stages and 16 discs from late-decay stages were calculated (Table 1).

**Table 1:** **Properties of Norway spruce (*Picea abies*) wood of different decay stages (early, intermediate and late) and the number of pooled discs used to prepare the inoculum (non-sterile sawdust) or the autoclaved sawdust for the microcosms**.

**Wood decay stage**	**C/N**	**Lignin (%)**	**Density kg/dm**	**^a^Dry matter (%)**	**^b^Water content (%)**	**No of pooled discs**	
						**inoculum**	**Substrate**
Early	352 ± 41	32 ± 5	0.42 ± 0.02	77 ± 0	30 ± 1	14	5
Intermediate	328 ± 63	37 ± 10	0.28 ± 0.01	59 ± 1	71 ± 3	12	5
Late	136 ± 42	51 ± 19	0.15 ± 0.02	21 ± 1	376 ± 16	16	5

The C/N ratio, lignin, diameter, and density (determined as described in Rajala et al., 2010) are the mean values (±SD) of 14 discs from early-decay stages, 12 discs from intermediate-decay stages and 16 discs from late-decay stages. Dry matter and water content were determined by drying fresh sawdust at 105°C for 24 h.

a Dry matter (%) was calculated as: weight of dry sawdust (g)/weight of wet sawdust (g) ^*^ 100.

b Water content (%) was calculated as: [weight of wet sawdust (g)—weight of dry sawdust (g)]/weight of dry sawdust (g) ^*^ 100.

Bark from the outermost layer of each frozen sample disc was removed with a flame-sterilized knife. Frozen discs were then drilled with a flame-sterilized and cooled bit (Ø = 10 mm) from the surface through the sapwood and heartwood. Shavings and sawdust of each decay stage were pooled into a single sample and stored in a plastic bag at −18°C prior to use as a fungal inoculum. A few active fungi might be suppressed during the processing of the samples, but species inhabiting dead wood in boreal forests are acclimated to varying temperatures and the fungal community obtained from the inocula was considered to reflect species composition in each decay phase.

The experimental growth medium (i.e., sawdust) was prepared by milling five discs from each decay stage that had been debarked and dried at 105°C for 48 h. Dry sawdust from each stage was pooled into a single sample and sterilized twice by autoclaving (121°C for 20 min) with a 3-day interval.

### Experimental set-up

The incubation took place in sterile 100-mL glass flasks containing different amounts of inoculum and autoclaved substrate (Table 2). Sterile distilled water was added to each flask to remoisten the culture to a dry matter content similar to that at sampling, i.e., 77% for early, 59% for intermediate and 21% for late decay stages (Tables 1, 2). Flasks were then sealed with rubber septa and a one-way stopcock was connected to a 50 mm needle. The stopcock system was designed to ventilate air and prevent anoxic conditions in the microcosm. The stopcock was opened only when the ventilation was performed after measurement of CO_2_ (see below). The air in the flasks was ventilated by connecting the capstock to a diaphragm vacuum pump (type N 022 AN.18, KNF Neuberger GmbH, Germany) for 2 min 30 s. The capstock was then closed and reopened to flush the flasks for 5 min with moist air previously channeled through an autoclaved vent filter (0.2 μm, Millipore, USA). The evacuation-flushing system prevented oxygen depletion and drought in the microcosms.

**Table 2:** **Amount of inoculum (non-sterile sawdust) or autoclaved sawdust in dry form added to 100-mL glass flasks to assess the effect of fungal diversity on the decomposition activities of Norway spruce sawdust in early, intermediate and late stages of decay**.

**Dilutions of the inoculum**	**^a^Inoculum (non-sterile sawdust)**		**Autoclaved sawdust**		**Added water**	**Total initial dry weight of the incubated sample**	**Initial dry matter content**
	**Decay stage**	**Amount (g, dm)**	**Decay stage**	**Amount (g, dm)**	**(ml)**	**(g, dm)**	**(%)**
Undiluted	Early	5.0	–	–	0	5.9	79
	Intermediate	3.8	–	–	0	3.8	59
	Late	1.4	–	–	0	1.4	21
Dilution 10^−1^	Early	0.54	Early	4.7	5.0	5.24	73
	Intermediate	0.4	Intermediate	3.5	3.0	3.9	52
	Late	0.15	Late	1.3	1.6	1.45	24
Dilution 10^−2^	Early	0.013	Early	4.7	5.0	4.71	66
	Intermediate	0.015	Intermediate	3.5	3.0	3.52	45
	Late	0.015	Late	1.3	1.6	1.32	23
Control (Ctrl)	–	–	Early	5.2	1.9	5.2	65
	–	–	Intermediate	3.8	3.5	3.8	46
	–	–	Late	1.5	5.7	1.5	21

The same amounts were used in a parallel experiment for the assessment of wood-degrading enzymes.

a Dry matter (dm) content of inoculum that was added as wet sawdust (water content according to field conditions, reported in Table 1).

### Preparation of microcosms and experimental design of decomposition study (experiment 1)

**Figure 1:**
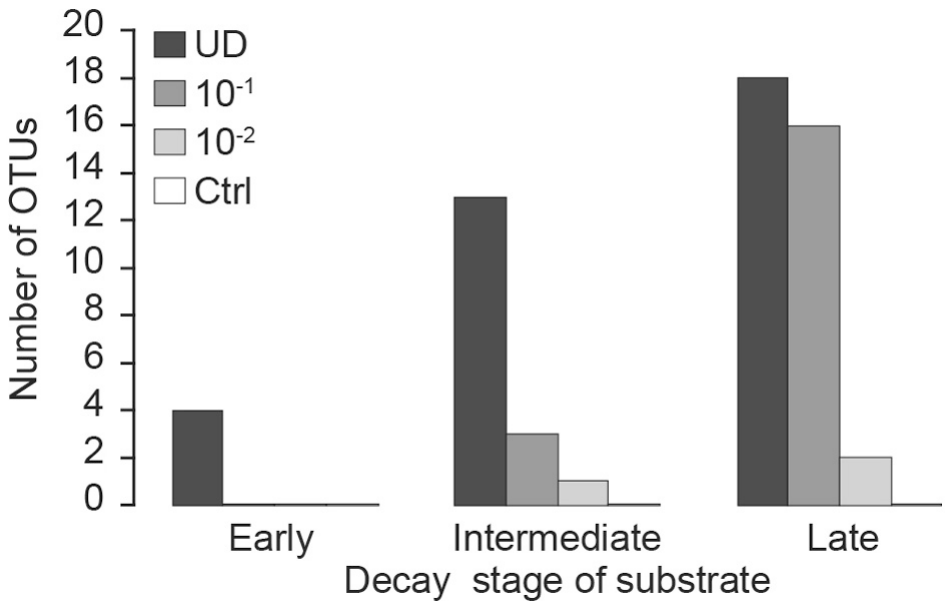
**Number of ITS1F-GC/ITS2 fungal DNA-derived DGGE bands (Operational Taxonomic Units; OTUs) in Norway spruce pooled substrate samples at the beginning of incubation under a range of inoculum dilutions (UD, undiluted fungal inoculum; dilution 10^−1^ g inoculum/g substrate (dm); dilution 10^−2^ g inoculum/g substrate (dm); Ctrl, autoclaved sawdust) for early, intermediate and late stages of decay**.

The effect of fungal diversity on respiration activity was assessed in microcosms by exposing the inoculum to a dilution procedure (Wertz et al., 2007). We tested four levels of fungal diversity (i.e., dilutions) on triplicate substrates of three different qualities (i.e., decay stages). Experimental conditions in microcosms were as follows: (1) undiluted (UD) inoculum prepared with non-sterile sawdust; (2) a dilution of approximately 1 g inoculum (dry matter, dm) per 10 g of substrate (dm) was prepared by first mixing non-sterile sawdust with sterile distilled water to create slurries with dry matter of 27% (i.e., early), 14% (i.e., intermediate), or 3% (i.e., late), then adding aliquots of 540 mg dm (early), 400 mg dm (intermediate), and 150 mg dm (late) to the corresponding dry substrate to yield a final inoculum dilution of 10^−1^ (Table 2); (3) a dilution of approximately 1 g inoculum (dm) per 100 g of substrate (dm) was prepared similarly, but slurry dry matter was 0.8% (early), 0.5% (intermediate) or 0.3% (late), and aliquots of 15 mg (dm) of each slurry were added to the corresponding dry substrate (Table 2); (4) autoclaved sawdust was used as a control (Ctrl). The initial fungal diversity of the microcosms was tested with the DGGE (Rajala et al., 2010 and description below) and the number of observed OTUs was used as a measure of fungal diversity. The test showed that the dilution procedure reduced the number of observed fungal OTU in the inocula (Figure 1). All treatments were performed in triplicate, yielding a total of 36 flasks (four levels of fungal diversity ^*^ three decay stages ^*^ three replicates = 36 flasks). Sealed flasks were incubated at 21°C in total darkness for 16 weeks. The cumulative CO_2_ was measured periodically (see below) and the airspace of the incubation flasks was ventilated immediately after CO_2_ measurement.

At the end of the incubation, the substrate from each replicate was pooled into a single sample and stored at –18°C prior to the extraction of total DNA.

### Measurement of CO_2_, evacuation and calculation of carbon loss

An air sample (100 μL) was collected from the sealed flask with a Hamilton Microfilter™ glass syringe, previously wiped with 95% ethanol, and injected manually into a gas chromatograph (Hewlett-Packard 6890, Finland) to analyse CO_2_ (assuming that most of the produced CO_2_ was transferred to the gas phase). The chromatograph was equipped with a capillary column (Agilent 19095P-MS6 HP-PLOT MoleSieve 5 Å, length 30 m and diameter 530 μm) and a temperature conductor detector at 250°C for detecting CO_2_. Helium was used as carrier gas at a split ratio of 10:1 and flow rate of 7.9 mL min^−1^. Injector and oven temperatures were 120 and 40°C, respectively.

The CO_2_ level was measured weekly at the beginning of the incubation and every 2 weeks after week five. Estimated carbon loss (*C loss*, %) from microcosms was calculated using cumulative CO_2_ at the end of the incubation following equations (1) and (2):

(1) $$\text{C loss}(\%)\;={\text{cumulative CO}}_{2}{\text{(g)/Max CO}}_{2}{\text{ (g) }}^{*}\;100$$

(2) $$\begin{array}{l} {\text{Max CO}}_{2}\text{(g)}={\text{mass sawdust (g) }}^{*}\text{ C}(\%)/100{\;}^{*}{\text{ M}}_{\text{CO}2}\\ {\text{ (g mol}}^{-1})/{\text{M}}_{\text{C}}\;({\text{g mol}}^{-1}) \end{array}$$

where *Max CO*_2_ is the maximum production of CO_2_ from each microcosm, *mass sawdus*t is the amount of sawdust in the flask (in dm), *C* is total carbon content in the sawdust, and *M*_*CO*2_ and *M*_*C*_ are the molecular masses of CO_2_ (44 g mol^−1^) and carbon (12 g mol^−1^), respectively.

### Preparation of microcosms and experimental design of the hydrolytic enzyme study (experiment 2)

The activity of several wood-degrading hydrolytic enzymes was measured from flasks incubated in parallel with the microcosms in Experiment 1. The experimental design was similar to that in Experiment 1 (i.e., four levels of fungal diversity ^*^ three decay stages ^*^ three replicates = 36 flasks). In this study, each flask was opened under sterile conditions after weeks 4, 6, 9, 11, 13, and 16 to collect about 1 g (wet weight, ww) of the substrate. Half of the sample was subjected immediately to enzyme activity assays and the other half was stored at −18°C prior to DNA extraction (samples from weeks 9 and 13).

### Extraction of enzymes and activity assays

Four glycoside hydrolases [β-glucuronidase (EC 3.2.1.31), β-xylosidase (EC 3.2.1.37), β-glucosidase (EC 3.2.1.21), and cellobiohydrolase (EC 3.2.1.37)] were recovered from the substrate samples following a recently described enzyme extraction method with slight modifications (Heinonsalo et al., 2012). The method is based on the recovery of extracellular enzymes by centrifuging a small amount of substrate (~155 mg, ww) placed into a centrifuge tube filter (0.45 μm pore size; 500 μL working volume; Costar^®^ Spin-X^®^ CLS8162; Corning Inc., NY, USA). The centrifugation speed was 15,700 *g* for 30 min. To obtain enough enzyme solution to run the assays, three sub-samples were prepared from each flask. The day before extraction, 200 μL sterile distilled water was added to each substrate sub-sample. After centrifugation, approximately 250 μL enzyme solution was recovered, pooled with that from the other three sub-samples, adjusted to a total volume of 2 mL with water and used directly for the activity assays. Once all the tubes were centrifuged, the inner filter tube was placed in an oven at 70°C for 48 h to measure substrate dry matter.

Hydrolytic enzymes were measured by a fluorimetric assay (Pritsch et al., 2004, 2011) and modified according to the requirements of this study. Three solutions were prepared: (1) incubation buffer (pH 4.5) containing 7.2 mM maleic acid, 7.3 mM citric acid, 10 mM boric acid, 10 mM Tris (2-amino-2(hydroxymethyl)-1,3-propanediol), and 48.8 mM sodium hydroxide; (2) different fluorogenic substrate solutions based on 4-methylumbelliferone (MU) for the detection of each glycosidic enzyme were prepared from 5 mM stock solutions in 2-methoxyethanol. The concentration of each fluorogenic substrate in the working solution was 1500 μM of MU-β-D-glucuronide (for detection of β-glucuronidase), 1500 μM of MU-β-D-xyloside (for detection of β-xylosidase), 1500 μM of MU-β-D-glucoside (for detection of β-glucosidase), and 1200 μM of MU-β-D-cellobioside (for detection of cellobiohydrolase); (3) the stop solution was 1 M Tris at pH 10-11 to enhance the fluorescence response. Triplicate assays were performed in black flat-bottom 96-well microplates, with each well-containing a reaction mixture of 50 μL enzyme solution, 50 μL incubation buffer and 50 μL of the respective fluorogenic substrate solution. Plates were incubated at 20°C on a microplate shaker for 30 min, except for β-glucosidase where 15 min was used. At the end of incubation, 150 μlL stop solution was added to all wells and the fluorescence response was measured with a Wallac 1430 Victor^3^ (PerkinElmer, Inc., USA) multilabel plate reader at an excitation wavelength of 364 nm and emission wavelength of 450 nm. Calibration wells were prepared by adding 100 μL stop solution, 100 μL incubation buffer and 50 μL stock solution mixed with water to give final MU concentrations of 0, 0.4, 0.8, 1.2, 1.6, and 2 μM.

Enzyme activities (EA) were expressed as picokatals per gram of substrate (in dm). A picokatal is one picomol of reacted fluorogenic substrate per second. *EA* were calculated using the equation (3). Total hydrolytic activity was estimated by summing that of the four measured enzymes.

(3) $$\text{EA }({\text{pkat g}}^{-1})=\text{(sample}-\text{negative control})/{({\text{a}}^{*}\text{t)}}^{*}{\text{V}}_{\text{a}}/{\text{V}}_{\text{s}}^{*}1/{\text{m}}_{\text{s}}$$

where *sample* is the fluorescence response (counts) of the enzyme solution and *negative control* is the response without enzyme (fluorogenic substrate without sample), *a* is the slope of the regression line of the calibration curve (counts/pmol), *t* is the incubation time (sec), *V*_*a*_ is the adjusted volume of the three sub-samples (2000 μL), *V*_*s*_ is the volume of the enzyme solution in the well (50 μL), and *m*_s_ is the sum of the extracted substrate mass of the three sub-samples (g, dm).

### Molecular analysis of the fungal community

Total DNA was extracted from substrates (100–150 mg, ww) at the beginning, after weeks 9 and 13 (only from the microcosms subjected to enzyme analyses) and at the end of incubation (week 16) using the NucleoSpin^®^ 96 Soil Kit (Macherey-Nagel, Germany). Extracted total DNA was amplified using polymerase chain reaction (PCR) with the GC-clamped internal transcribed spacer 1 (ITS1F) primer (Gardes and Bruns, 1993) and the ITS2 primer pair (White et al., 1990) typical of fungal ribosomal DNA. PCR was performed in a 50 μL reaction containing 25 μL MyTaq™ HS Red Mix (which included reaction buffer, dNTP, DNA Polymerase and loading dye; Bioline, Germany), 1 μL each 25 μM primer, 2.5 μL template and 20.5 μL sterile distilled water. The thermal profile for the PCR was: 1 min at 95°C for initial denaturation, 34 cycles of denaturation for 15 s at 95°C, annealing for 15 s at 58°C and extension for 10 s at 72°C, and a final extension of 72°C for 10 s. Equal concentrations of PCR products were resolved by denaturing gradient gel electrophoresis (DGGE) as described in Rajala et al. (2010). Briefly, the denaturing gradient in an acrylamide gel was 18–58% and running conditions were 75 V at 60°C for 16 h. Gels were stained with SYBR^®^ Gold (Molecular Probes, Eugene, Oregon) and visualized with blue light on a SafeImager™ transilluminator (Invitrogen, Carlsbad, California). The DGGE gels were analyzed using GelCompar II software (Applied Maths BVBA, Belgium). The bands at the same position in the DGGE gel were considered the same operational taxonomic unit (OTU). Thus, this step generated a presence–absence matrix of fungal taxa (OTUs) in each microcosm and we used the number of the detected OTUs as a measure of fungal diversity in this experiment.

### Statistical analysis

We analyzed the effects of inoculum dilution (undiluted, 10^−1^ and 10^−2^) and decay stage on CO_2_ production and enzymes activities measured from samples during the incubation period using the methods of longitudinal data analysis (Diggle et al., 2002). We also performed some additional analyses of cumulative CO_2_ production during the entire incubation period and on EA at the end of the experiment (week 16) using ANOVA, where longitudinal data analysis was not needed. In addition, we tested correlation between the number of observed OTUs and the cumulative CO_2_ production at the end of the experiment.

In the longitudinal analyses of consecutive weekly CO_2_ production and log-transformed total hydrolytic EA of the samples, we used a mixed model where replicates (r) represented random effects [~ N(0, σ^2^_*r*_)]. The fixed part of the model consisted of the decay stage and dilution factors, and their interaction, i.e., the effects we were interested in. Serial correlation of repeated measurements in the time series of replicates was treated with a continuous autoregressive covariance structure AR(1).

Residuals of the fitted models were inspected visually for normality and homoscedasticity. In all cases, we found that the residuals of the CO_2_ fit were normally distributed by fitted values, and the distributions were also normal and fairly similar by explanatory variables. Random effects were distributed approximately normally as well. To fulfil these requirements for the total hydrolytic EA, we converted them to a log-scale.

Strong interactions in the fixed part of the model can make it difficult to interpret the model. Therefore, if we found significant interactions between the fixed effects, we simplified the model by either regrouping the dilution and decay stage to a single factor, or by fitting the model for dilution factor by the decay-stage factor, or the other way around.

We also investigated the trends of log-transformed total hydrolytic enzyme activity, and the ratio of hydrolytic activity to CO_2_ production during the incubation period. In these cases, we added a slope parameter for incubation week to the fixed part of the model. The trends for these variables were tested for each decay stage separately, so that we allowed the trend to vary by dilution. For the ratio of hydrolytic activity and CO_2_ production, the results are presented for the early decay stage by dilution.

Longitudinal analyses were implemented using the lme function of the nlme library of R, using the maximum likelihood method. Parameter significance was estimated by the lme-function.

When a time-series approach was not needed, we used a type II ANOVA with interaction between the decay stage. These analyses were conducted using the ANOVA function in the car-library (Fox and Weisberg, 2011) of R (R Development Core Team, 2011). The same principles of examining the residuals and splitting the model into parts if interactions were present, were applied as with the longitudinal approach. Pairwise comparisons were made with the Tukey Honestly Significant Difference (HSD) test.

Fungal community composition (i.e., the presence/absence of OTUs) was visualized by non-metric multidimensional scaling (NMDS) using metaMDS of the vegan library (Oksanen et al., 2008). We generated a dissimilarity matrix (*n*_sample_^*^ *n*_sample_) of Bray-Curtis coefficients and used NMDS to create a graph where the distances between points (representing samples) corresponded as closely as possible to the original dissimilarity matrix. Arrows that indicate the direction of maximum correlation of cumulative CO_2_ and OTU number with the ordinated samples were superimposed onto the ordination graph. The significance of these correlations was assessed with a permutation test implemented in the envfit function of vegan library (Oksanen et al., 2008). The NMDS was performed separately for each incubation time. The analyses made for week 0 and 16 corresponded to the experiment where flasks were closed during incubation (Experiment 1), whereas those made for week 9 and 13 corresponded to the microcosms in which flasks were opened to collect samples (Experiment 2).

## Results

### Effects of substrate and reduced diversity on decomposition

**Figure 2:**
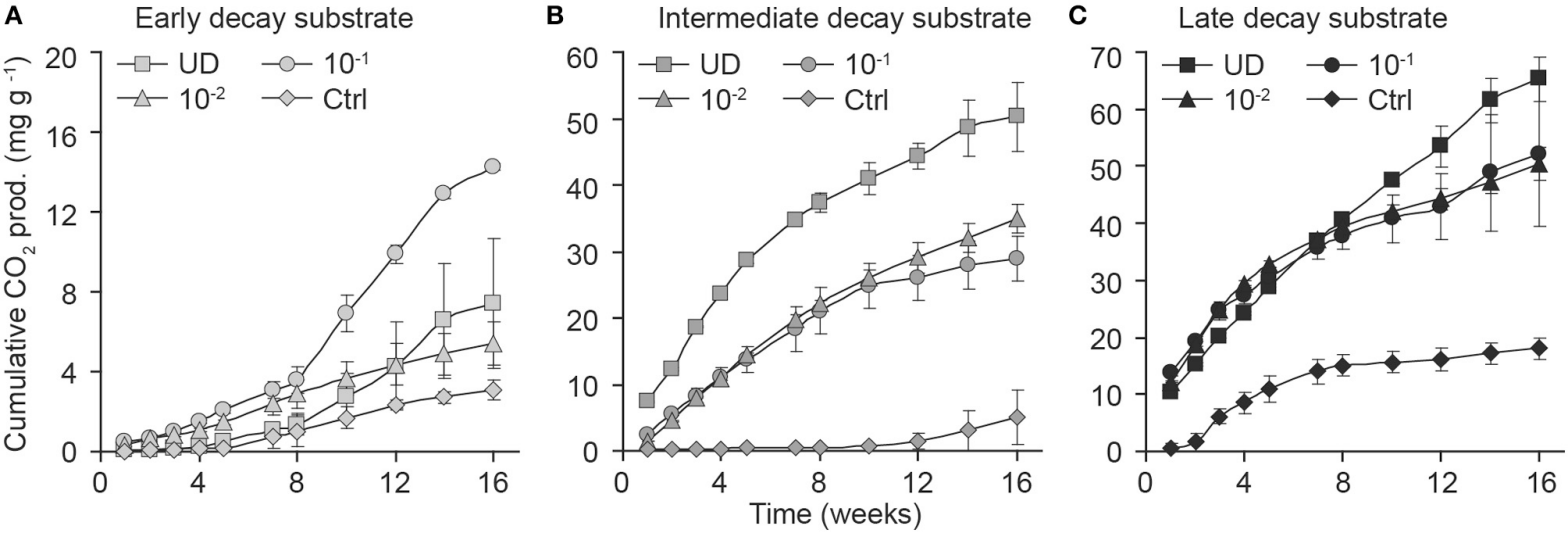
**Effect of dilution on the production of CO_2_ (cumulative mg of CO_2_ per g of substrate; in dm) within early- (A), intermediate- (B), and late- (C) decay stages**. Dilution of fungal diversity is shown by symbols as undiluted fungal inoculum (squares), dilution 10^−1^ g inoculum/g substrate (circles), dilution 10^−2^ g inoculum/g substrate (triangles), and controls (diamonds). Error bars indicate the *SD* of the mean for observations and were calculated for each decay stage and dilution separately.

The wood decay stage was an important factor to explain CO_2_ production during the experiment (Figure 2, Supplementary Material). When we analyzed the consecutive CO_2_ observations and their association with the decay stage with longitudinal analysis, we found that late- and intermediate-decay substrates were associated with significantly higher CO_2_ production rates than early-decay substrates (*t* = 8.0, *df* = 17, *p* ≤ 0.001, and *t* = 5.7, *df* = 17, *p* < 0.001, respectively). This effect was present when we analyzed each inoculum separately (*p* < 0.02 for decay-stage differences within inocula), although the 10^−1^ inoculum responded significantly differently from other inocula in the full model with interactions.

**Figure 3:**
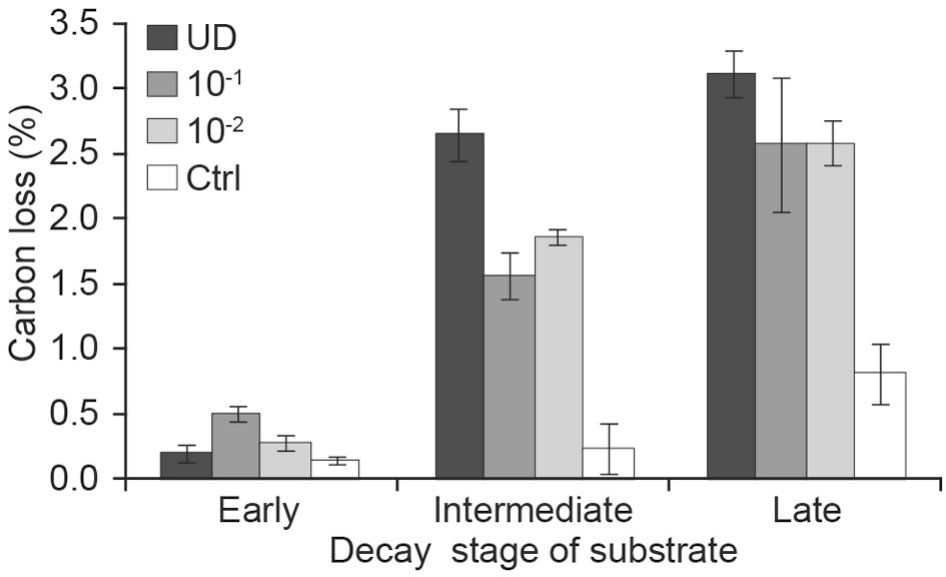
**Cumulative carbon loss (means of three replicates) during the incubation of early, intermediate and late stages of decay and receiving various dilutions of fungal inoculum [UD, undiluted; dilution 10^−1^ g inoculum/g substrate (dm); dilution 10^−2^ g inoculum/g substrate (dm); Ctrl, autoclaved sawdust]**.

When we analyzed the effect of inoculum (which had different number of fungal OTUs) according to decay stage with longitudinal analysis, we found that the weekly CO_2_ production of diluted 10^−1^ g and 10^−2^ g inocula differed from that of undiluted inoculum in intermediate-decay substrates (*t* = −5.5, *p* = 0.002 and *t* = −3.9, *p* = 0.008, respectively). The ANOVA of cumulative CO_2_ at week 16 supported these findings. When fungal diversity (OTU) was added to the previous model, it was also significant (*F* = 4.4, *df* = 1, *p* = 0.05). After 16 weeks, the cumulative production of CO_2_ from undiluted inoculum in late-decay substrates reached 65 mg CO_2_ g^−1^ substrate (dm), corresponding to a C loss of 3.1%, whereas C loss from undiluted intermediate- and early-decay substrates were 2.6 and 0.2%, respectively (Figures 2C, 3). At the end of the experiment, the cumulative CO_2_ production was correlated with the observed number of OTUs (*r* = 0.83, *p* < 0.001).

### Effects of substrate and reduced diversity on enzyme activity

**Figure 4:**
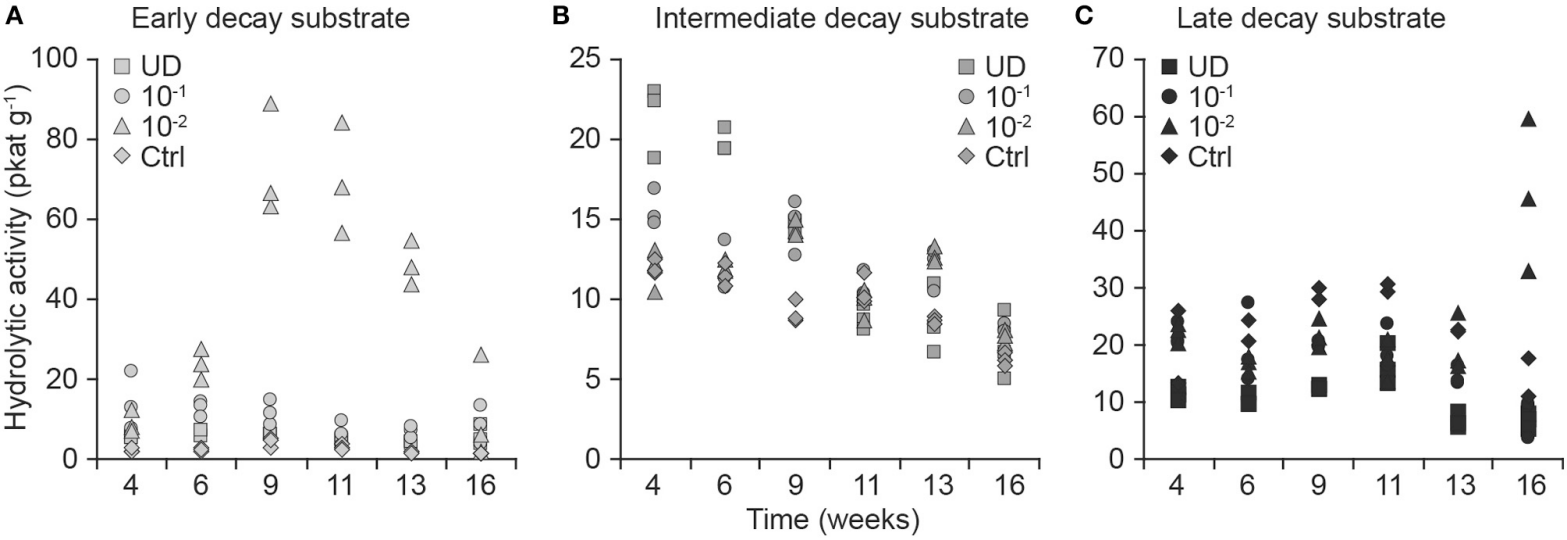
**Effect of fungal community dilution on total hydrolytic activity (pkat/g of substrate; in dm) as the sum of 1,4-β-glucuronidase, 1,4-β-xylosidase, cellobiohydrolase, and 1,4-β-glucosidase from early-decayed (A), intermediate-decayed (B), and late-decayed substrate (C)**. The dilution of fungal diversity is shown by symbols as undiluted fungal inoculum (squares), dilution 10^−1^ g inoculum/g substrate (circles), dilution 10^−2^ g inoculum/g substrate (triangles), and controls (diamonds).

Differences in total hydrolytic EA depended on the substrate, therefore, we performed the analyses by substrate and by dilution (Figure 4).

When we analyzed the consecutive total hydrolytic activity of replicates with longitudinal analysis, we found that it was higher in preparations containing the 10^−2^ dilution than in the undiluted inoculum in early- (*t* = 3.6, *df* = 5, *p* = 0.02) and late-decay substrates (*t* = 6.0, *df* = 6, *p* = 0.001). Moreover, the hydrolytic activities were also significantly different between early decayed and other substrates in undiluted inocula (both *t* > 4.0, *df* = 6, *p* < 0.005) and in the 10^−1^ dilution (both *t* > 3.2, *df* = 5, *p* < 0.03).

When we analyzed the total hydrolytic enzyme activity measurements at the end of the experiment with ANOVA, we also found that the activities depended on the dilution-substrate combination (interaction *F* = 10.2, *df* = 6, *p* < 0.001). We then grouped the inoculum and substrate into a compounded factor, performed an ANOVA and conducted a pair-wise comparison of factors, which showed that the mean hydrolytic activity of the most diluted (10^−2^) inoculum of the late-decayed substrate was greater than that in any of the other inoculum decay-stage combinations (*p* < 0.0001 for all pairs).

Furthermore, the number of fungal OTUs appeared to influence the enzyme activity at the end of the experiment (*F* = 14.2, *df* = 1, *p* = 0.09).

In all dilutions of the intermediate-decay inoculum, a negative trend in total hydrolytic activity was recorded toward the end of the incubation (Figure 4B). According to the longitudinal analysis, the higher intercept and stronger negative trend of hydrolytic activity in undiluted inoculum than in either of the diluted inocula (the model intercept differences in comparison to the undiluted inoculum both had *t* < −3.0, *df* = 6, *p* < 0.02 and for the trend coefficient, they had *t* > 3.4, *df* = 42, *p* < 0.001, respectively) were coincident with the measured respiration rates that started at the highest values (*t* < −3.9, *df* = 6, *p* < 0.01) and then decreased the quickest (*t* > 2.3, *df* = 42, *p* < 0.02) in the undiluted inoculum in comparison with other dilutions in the intermediate-decay substrate (Figures 2B, 4B).

**Figure 5:**
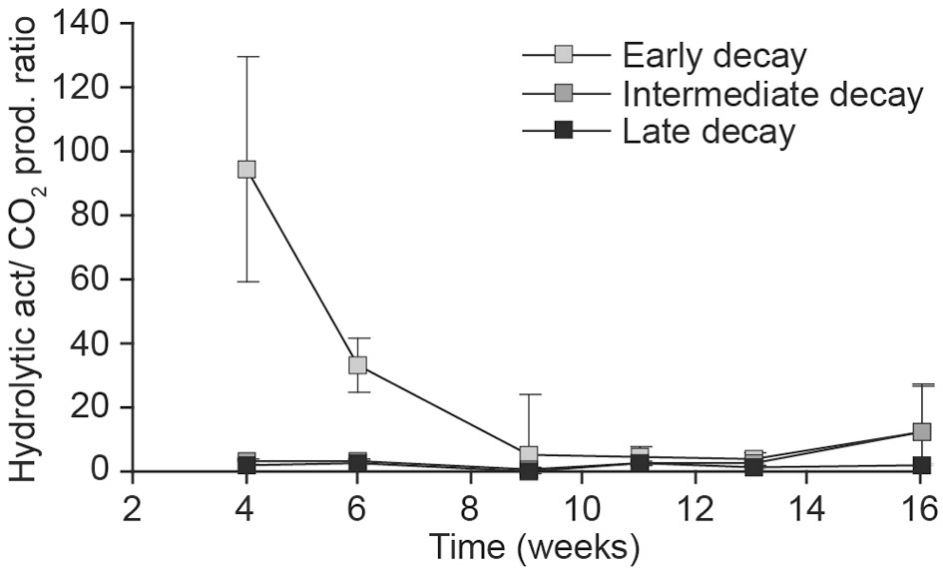
**Relationship of carbohydrate degradation (pkat/g) and weekly CO_2_ production (mg/g substrate, dm) in the treatments receiving undiluted fungal inoculum of early- (light gray), intermediate- (dark gray), and late- (black) decay substrate during the 16-week incubation**.

The ratio of hydrolytic activity to respiration (Hydrolytic activity/CO_2_) clearly decreased toward the end of the incubation in the early decay substrate that received undiluted (*t* = −3, 4, *df* = 13, *p* = 0.005), as well as diluted inocula (10^−1^ dilution: *t* = −2.9, *df* = 9, *p* < 0.02) (Figure 5). The same phenomenon did not occur in more decayed substrates (data not shown).

### Fungal community structure and respiration activity in different decay stages

**Figure 6:**
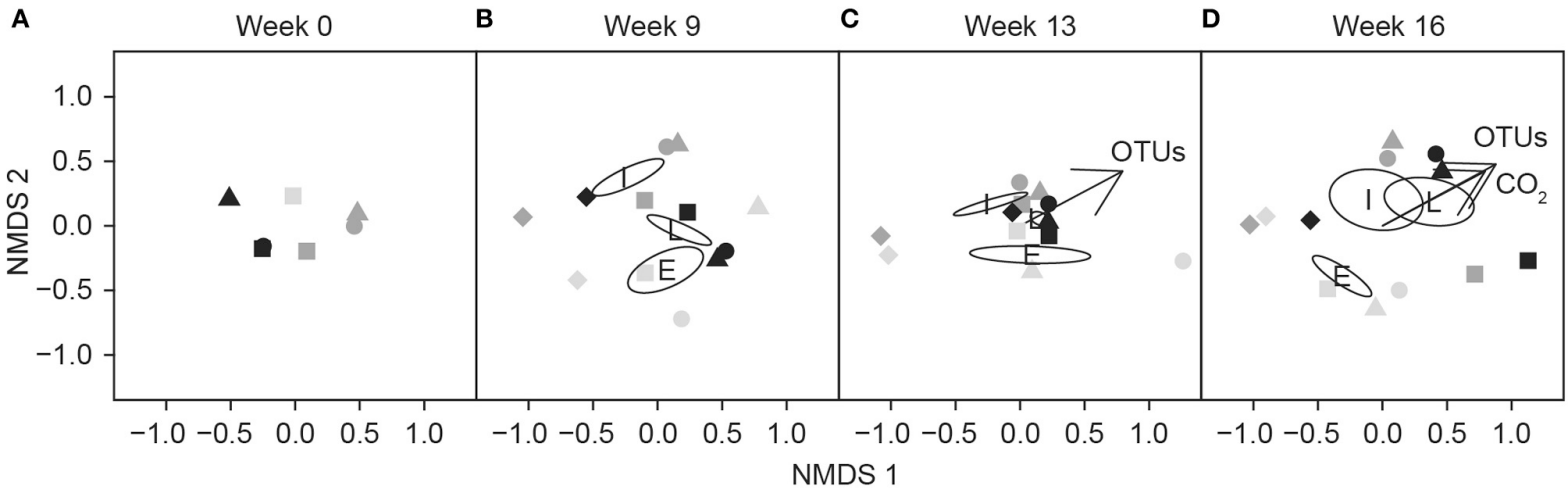
**Non-metric multidimensional scaling (NMDS) ordination showing the separation of fungal communities at the beginning (A), after 9 weeks (B), 13 weeks (C), and 16 weeks (D) in the different dilutions or controls (autoclaved sawdust) according to the decay stage of substrate**. Symbols represent the dilution of the fungal community: undiluted (squares), dilution 10^−1^ g inoculum/g substrate (circles), dilution 10^−2^ g inoculum/g substrate (triangles) and controls (diamonds). The intensity of the symbol indicates the decay stage. Vectors indicate the direction of maximum correlation of CO_2_ production and number of OTUs with ordinated samples (*p* < 0.01). Ellipses indicate the mean location of each decay stage: E, early; I, intermediate; and L, late.

Fungal communities of different decay stages differed at the start of the experiment (Figure 6A) and community structures changed during the incubation period (Figure 6). In conjunction with observed differences between undiluted and diluted fungal communities in the intermediate decay substrate (Figure 6B), we measured higher respiration activity in the microcosm prepared with the undiluted inoculum, especially during the first 6 weeks (Figure 2B). In the early decay substrate, the slightly diluted (10^−1^) fungal community, which had the highest respiration rate (Figure 2A), appeared to deviate from all other communities after 13 weeks of incubation (Figure 6C).

At the end of the experiment, cumulative CO_2_ (*r* = 0.91, *p* < 0.001) and the number of OTUs (*r* = 0.87, *p* < 0.001) increased in the communities composed of intermediate- and late-decay stage species, as shown by the linear fit superimposed on the NMDS graph (Figure 6D).

## Discussion

### Effects of fungal diversity on wood decomposition

The main findings of the experiment confirmed our hypothesis that fungal diversity affects decomposition rate and that resistance of the decomposition process to the loss of diversity depends on the decomposition stage of woody substrates. We used inocula that originated from actively decaying wood, where the number of OTUs is higher than in previous experiments using selected species from pure cultures (Setälä and McLean, 2004; Tiunov and Scheu, 2005). These studies with communities created by combining cultured fungal species have shown how the decomposition rate becomes asymptotic with relatively few decomposer species, and our results on late-decay communities with initially high number of fungal OTUs support this finding. However, we observed that the functioning of the decomposers of the intermediate-decay stage, where the initial number of OTUs was lower than in the late-decay wood, was less resistant to the loss of diversity. In the intermediate-decay stage, decomposition activity was highest in non-diluted communities and the experimental dilution of the fungal community was associated with a lower respiration rate.

This study showed that CO_2_ production, as an indicator of decomposition, increased in relation to the decay stage of Norway spruce wood and peaked during the late-decay stage, where fungal diversity (measured as the number of observed OTUs) was also maximal. The observed increase in decomposition activity with increasing OTU diversity is in agreement with our hypothesis. Earlier studies, where decay models were fitted to empirical field data, predicted that the rate of decay is highest during intermediate phases of decomposition (Harmon et al., 2000; Mäkinen et al., 2006; Tuomi et al., 2011), which coincides with the period when white- and brown-rot fungi are at their peak prevalence (Rajala et al., 2011, 2012). However, we suggest that decomposition activity is also maintained in the late-decay stage wood that might be under-represented in previous studies (i.e., they used a relatively small number of late-decay samples, which are difficult to date). In addition, our results indicate that lowering fungal diversity, especially in the intermediate-decay stage, has a direct impact on fungal community function, and thereby on the decomposition of woody litter.

We showed that in the most diverse fungal community inhabiting the late-decay substrate, lowering the diversity with dilution of the inocula had only a slight impact on decomposition rate. This suggests that the community that remained after dilution was sufficiently diverse to provide the same ecological function as that of the undiluted system, which agrees well with earlier studies of the diversity-function relationship in soil microbes (Wertz et al., 2006), whereas the less diverse communities were more affected by random selection. Consequently, we predict a stronger resilience of the diverse fungal community found in advanced stages of wood decay.

In the less diverse community inhabiting the early decay substrate, a 10^−1^ dilution caused an unexpected result in that CO_2_ production increased. It appears that loss of diversity in a low-density community affects competitive interactions and decomposition in unpredictable ways. The response is highly sensitive to the activity of the remaining species and the functional redundancy in the fungal community of early-stage decomposers is lower than that found in later stages.

The use of indigenous fungal communities as inocula to study the relationship between diversity and function of terrestrial fungal communities is novel and was applied for the first time to wood-inhabiting fungi in this study. Previous laboratory studies have assessed the ability of cultured fungi to degrade woody substrates (Boddy et al., 1989; Tiunov and Scheu, 2005; Toljander et al., 2011) and compete with natural wood-inhabiting fungi (Holmer and Stenlid, 1997; Holmer et al., 1997). According to the method proposed by Wertz et al. (2006), the dilution procedure reduced the number of fungal taxa detected in the inoculum. Prior to our experiment, we tested different ratios of dilutions and found that a relatively high (1/100) dilution is needed for a remarkable reduction of OTUs in highly diverse inocula. However, for a moderate reduction in the OTU diversity, we chose to apply a dilution ratio (1/10) that was optimal for the intermediate decay stage, but compromised late decay, where the resulting reduction in OTU diversity was only slight.

As expected, the number of detected fungal species increased in all treatments with the progression of the incubation, since the applied PCR-DGGE method cannot identify infrequent fungi and only after replication of DNA during the incubation they became visible. Our sterilization procedure of the control substrate was apparently sufficient to destroy most of the fungal DNA, as OTUs were not detectable via DGGE at the start of the experiment (Figure 1). However, slight CO_2_ production during the incubation indicated that some microbes survived autoclaving (Figure 2), as it is known that some spores might be resistant to this form of sterilization (Cheng et al., 2008). This was the reason to detect a few fungal OTUs in the controls after 9 weeks (data not shown). However, NMDS showed the community structure in controls to be distinct from that of other microcosms. This suggests that the key species involved in decomposition did not survive autoclaving, although we cannot totally deny that some species originated from autoclaved sawdust. However, CO_2_ production in controls was far lower than in any of the treatments and therefore we believe that effect of contaminant species in dilution treatments is minor. The fact that sterilization of wood, soil or other substrate by autoclaving is practically impossible should still be considered in this kind of dilution procedures.

### Enzyme activity during wood decomposition in response to fungal diversity

Contrary to our hypothesis, the most diluted (10^−2^) inoculum was associated with a higher activity of hydrolytic enzymes than treatments of the same substrate receiving inoculum with a higher fungal richness (Figure 4). This might be explained by less competition in the diluted communities involving species specializing in the production of carbohydrate-degrading enzymes (e.g., brown- and soft-rot fungi). We also found that the ratio of hydrolytic activity and production of CO_2_ per week in early-decay substrates was initially high and gradually decreased. The negative slope of the relationship between hydrolytic activity and weekly CO_2_ production suggests that fungal taxa that are active during the early phases of decomposition must produce hydrolytic enzymes to release the wide variety of carbohydrates that are metabolized in later stages.

The enzyme recovery method, based on a filter centrifugation approach, has been validated in agar media supplemented with different organic products (Heinonsalo et al., 2012), but has never been applied to a more complex substrate such as one composed of decomposing spruce wood. A standard and universal protocol for extracting and measuring enzymes from environmental samples does not exist (Baldrian, 2009). Common practice is to soak the samples in buffer (e.g., acetate or sulfate buffers) and extract the enzymes by agitating the slurry, but such treatment might negatively affect EA (Vepsäläinen, 2001). In our study, the level of enzyme activity was in the range 10–100 pkat g^−1^ dry substrate. Previous studies that reported activities as high as 1000 pkat g^−1^ (dm) in Norway spruce needles used pure fungal inoculum from a cultured isolate (Žifèáková et al., 2011). Our results indicate that extracellular EA in spruce wood that has not received a pure and concentrated inoculum are much lower. It is known that spruce wood is a complex substrate for the recovery of wood-degrading enzymes, since water-soluble constituents such as phenols, sugars, organic acids, xylo-oligosaccharides and proteins all act as inhibitors of hydrolytic enzymes (e.g., Lagaert et al., 2009; Kim et al., 2011). Nevertheless, the effect of wood extracts on enzyme recovery in this study was expected to be minimal as was previously reported by Valentín et al. (2010).

## Conclusions

Dilution of fungal inocula recovered from *Picea abies* logs in three different decay stages revealed a stage-dependent response in decomposition rate. Reduced fungal diversity was associated with lower respiration rates during the intermediate stages of decay, but no diversity effects were detected in later stages, where initial number of detected species was high. This suggested that the highly diverse community of the late-decay stage was more resistant to the loss of diversity than less diverse communities of early decomposers. In early-decay communities, dilution caused unexpected changes to the decomposition process, probably due to the strong stochastic effect of dilution on less diverse communities. The results of this study also showed that the decomposition rate and the fungal diversity increased as decay advanced. We suggest that fungal activity and the functional redundancy of the fungal community in decaying wood increase during the fungal succession from intermediate- to late-decay stages.

### Conflict of interest statement

The authors declare that the research was conducted in the absence of any commercial or financial relationships that could be construed as a potential conflict of interest.
