# Supplementary material for: Loss of diversity in wood-inhabiting fungal communities affects decomposition activity in Norway spruce wood
Source: Front Microbiol. 2014 May 20;5:230. doi: 10.3389/fmicb.2014.00230 (PMC4032996; doi:10.3389/fmicb.2014.00230)
Supplement: Supplementary file 1 [file DataSheet1.PDF]

Appendix 1. Weekly production of CO_2 (mg of CO_2 per g of substrate; in dm) of each decay stage of the inoculum and the dilutions.

Values indicate the mean of three replicates ± standard deviation.

		Time (weeks)										
**Decay stage**	**Dilutions of the inoculum**	**1**	**2**	**3**	**4**	**5**	**7**	**8**	**10**	**12**	**14**	**16**
Early	Undiluted	0.1 ± 0	0 ± 0	0.1 ± 0	0.1 ± 0	0.2 ± 0.1	0.6 ± 0.2	0.3 ± 0.2	1.4 ± 0.9	1.5 ± 1.1	2.3 ± 1.4	0.9 ± 0.5
	Dilution 10^{-1}	0.5 ± 0.1	0.2 ± 0	0.3 ± 0	0.5 ± 0	0.6 ± 0.1	1.0 ± 0.2	0.5 ± 0.3	3.3 ± 1.6	3.0 ± 0.5	3.0 ± 0.6	1.3 ± 0
	Dilution 10^{-2}	0.3 ± 0.1	0.3 ± 0.1	0.2 ± 0.1	0.2 ± 0.1	0.4 ± 0.2	1.0 ± 0.6	0.4 ± 0.1	0.8 ± 0.2	0.7 ± 0.3	0.5 ± 0.2	0.5 ± 0.1
	Control	0 ± 0	0 ± 0	0 ± 0	0 ± 0	0.1 ± 0.1	0.5 ± 0.5	0.3 ± 0.2	0.6 ± 0.2	0.7 ± 0.4	0.4 ± 0.3	0.3 ± 0.2
Intermediate	Undiluted	7.4 ± 0.2	4.9 ± 0.1	6.3 ± 0.1	5.0 ± 0.1	4.9 ± 0.2	6.2 ± 0.5	2.5 ± 0.5	3.7 ± 1.2	3.3 ± 0.8	4.3 ± 3.0	1.6 ± 1.0
	Dilution 10^{-1}	2.4 ± 0.1	3.0 ± 0.1	2.8 ± 0.8	2.7 ± 0.5	2.6 ± 0.7	4.6 ± 2.0	2.7 ± 0.2	3.8 ± 0.2	1.2 ± 0.5	1.9 ± 0.1	0.9 ± 0.1
	Dilution 10^{-2}	1.4 ± 0	3.1 ± 0.1	3.5 ± 0.1	2.8 ± 0.1	3.5 ± 0.3	5.4 ± 0.5	2.4 ± 0.2	4.0 ± 1.0	3.0 ± 1.7	2.8 ± 0.5	3.0 ± 0.5
	Control	0.1 ± 0	0.1 ± 0	0.1 ± 0	0.1 ± 0	0.1 ± 0	0.1 ± 0	0.1 ± 0	0.2 ± 0	0.8 ± 1.1	1.5 ± 2.0	2.0 ± 1.7
Late	Undiluted	10.3 ± 0.3	4.9 ± 0.1	5.0 ± 0.1	4.0 ± 0.2	4.4 ± 0.7	8.0 ± 0.7	3.8 ± 0.5	7.1 ± 0.8	5.8 ± 3.5	8.1 ± 0.7	3.8 ± 0.5
	Dilution 10^{-1}	13.8 ± 0.6	5.5 ± 0.5	5.4 ± 0.6	2.7 ± 0.1	3.2 ± 0.3	5.0 ± 0.4	2.2 ± 0.5	3.0 ± 1.8	2.0 ± 1.6	6.0 ± 4.7	3.3 ± 2.7
	Dilution 10^{-2}	12.2 ± 0.2	6.4 ± 0.5	6.2 ± 0.2	4.6 ± 0.3	3.4 ± 0.2	4.5 ± 0.3	2.2 ± 0.4	2.7 ± 0.4	2.1 ± 0.8	3.1 ± 1.2	3.1 ± 1.0
	Control	0.4 ± 0.6	0.8 ± 0.9	3.0 ± 3.2	1.6 ± 1.4	1.6 ± 1.4	2.1 ± 1.7	0.8 ± 0.6	0.4 ± 0.3	0.4 ± 0.3	2.6 ± 2.8	2.1 ± 2.1
